# A Modified Arthroscopic Approach for Localized Pigmented Villonodular Synovitis in the Posterior Knee Compartment

**DOI:** 10.1016/j.eats.2025.103573

**Published:** 2025-05-07

**Authors:** Kengo Harato, Shu Kobayashi, Tasuaki Matsumoto, Kazuya Kaneda

**Affiliations:** a Sports Medicine Research Center, Keio University, Yokohama, Japan; b Department of Orthopedic Surgery, Keio University School of Medicine, Tokyo, Japan

## Abstract

Localized pigmented villonodular synovitis of the posterior knee compartment presents a surgical challenge due to limited arthroscopic access and the complex anatomy of the posterior knee. We describe a modified arthroscopic approach that enhances visualization and facilitates efficient resection by enlarging the posteromedial portal. This technique allows simultaneous use of the arthroscope and surgical instruments within the same portal, optimizing maneuverability and precision. The procedure involves careful portal placement, strategic use of a 70° arthroscope, and a combination of curettage, shaving, and radiofrequency ablation to achieve complete resection while minimizing iatrogenic injury. Compared to traditional approaches using both posteromedial and posterolateral portals, our method is less invasive yet provides adequate exposure for thorough lesion removal. Additionally, resection performed solely from anterior portals has been reported, but it frequently results in incomplete excision due to restricted access. This technique offers distinct advantages, including improved visualization, efficient instrument handling, and reduced operative time, making it particularly suitable for novice surgeons. By refining arthroscopic accessibility to the posterior knee compartment, this approach represents a safe, effective, and reproducible method for managing localized pigmented villonodular synovitis with minimal morbidity.

Pigmented villonodular synovitis (PVNS) is a rare, benign proliferative disorder of the synovial membrane, characterized histologically by hemosiderin deposition, multinucleated giant cells, and hypervascularization. PVNS can present in either a diffuse or localized form, with the latter (localized PVNS, or LPVNS) being less aggressive but still capable of causing significant joint dysfunction.^1^^,^^2^ While the knee is the most frequently affected joint, involvement of the posterior compartment remains relatively uncommon.^3^ The management of LPVNS in the posterior knee compartment presents distinct challenges due to limited arthroscopic access and the close proximity of neurovascular structures.^4^ Historically, open synovectomy has been considered the gold standard for complete resection; however, arthroscopic techniques have evolved to provide minimally invasive alternatives with lower morbidity and faster recovery. Although some reports describe a 2-portal approach using both posteromedial and posterolateral portals, these methods can be technically demanding and more invasive.^5^ Conversely, an alternative approach involving resection via anterior portals alone has been described, although it may lead to incomplete excision due to limited access.^4^ We describe a modified arthroscopic approach that utilizes an enlarged posteromedial portal, allowing for better visualization and effective lesion removal. By enabling simultaneous access to surgical instruments and the arthroscope through a single portal, this technique facilitates thorough synovectomy while maintaining a minimally invasive approach (Video 1). We discuss the surgical steps while emphasizing the advantages of this method compared to conventional techniques.

## Surgical Technique

**Fig 1:**
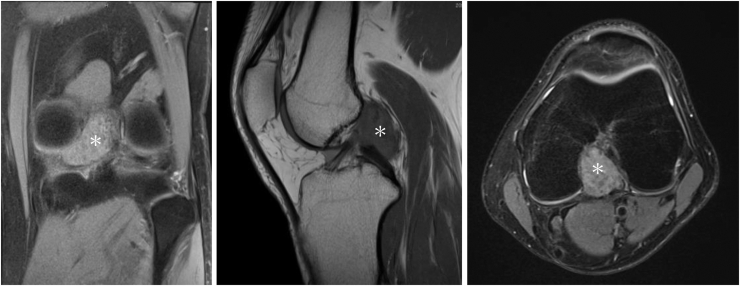
Preoperative magnetic resonance imaging of the left knee showing the localized pigmented villonodular synovitis (localized pigmented villonodular synovitis: white asterisk) in the posterior knee compartment, highlighting the extent and anatomic location of the lesion.

**Fig 2:**
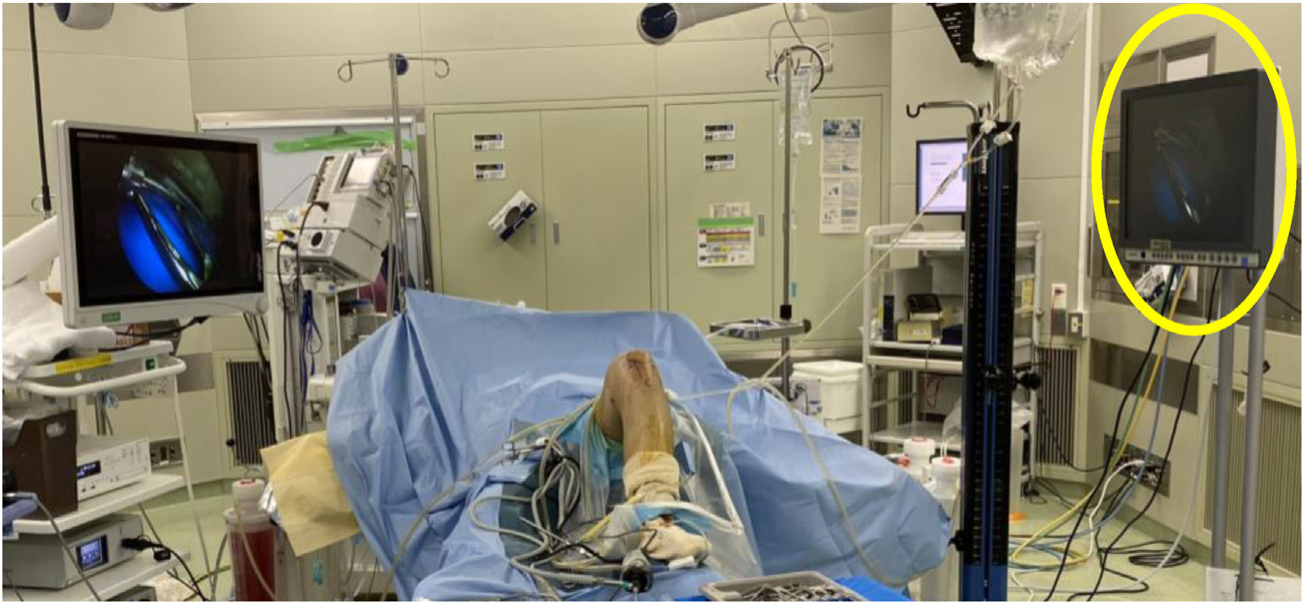
The patient is positioned supine with a standard leg holder (Mizuho), allowing full range of motion. Surgical setup illustrating the placement of an additional monitor (yellow circle) on the affected (left knee in this case) side to enhance visualization during arthroscopy.

**Fig 3:**
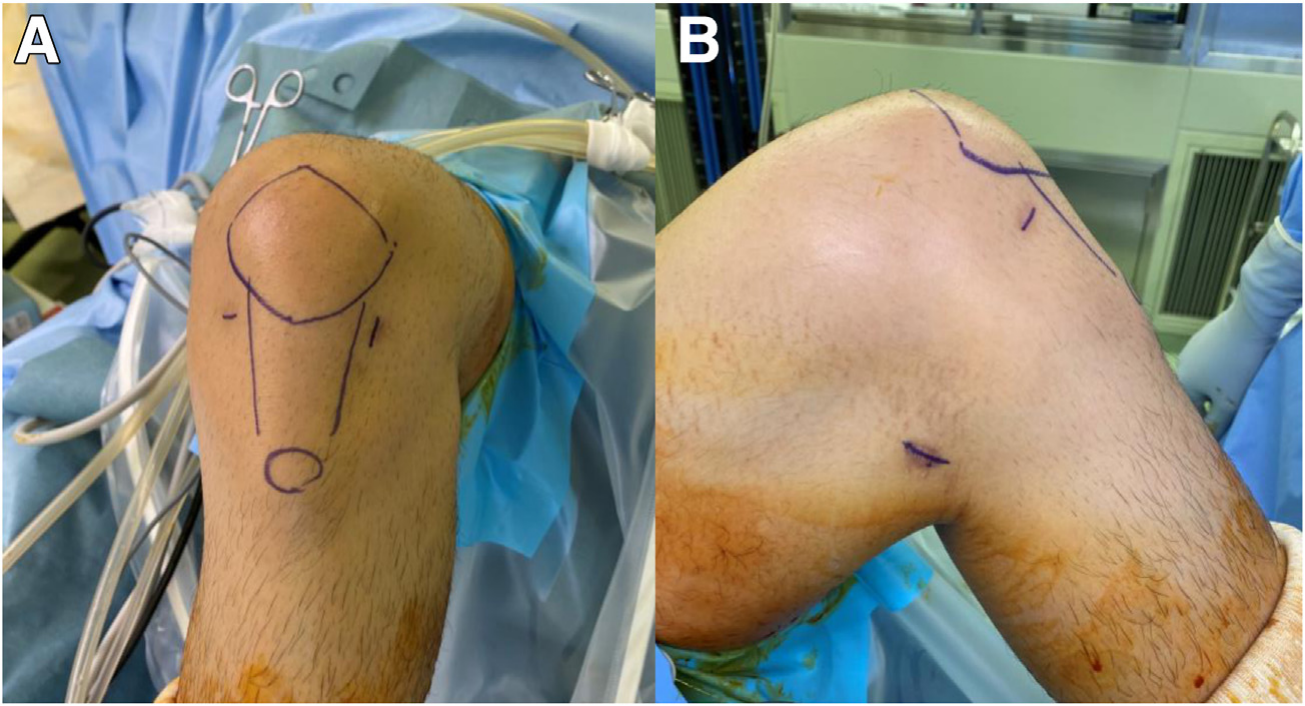
(A) Standard knee arthroscopy initiated through anterolateral (viewing) and anteromedial (working) portals. (B) Creation and enlargement of the posteromedial portal to facilitate instrument maneuverability. To establish the posteromedial portal, a 70° arthroscope through the viewing portal and a 21-gauge needle are used to confirm the precise location, which is the soft spot near the posteromedial margin of the medial femoral condyle.

The detailed surgical steps are described in Table 1. For preoperative preparation, magnetic resonance imaging is performed to evaluate the location and size of LPVNS (Fig 1). LPVNS of the posteromedial knee compartment is the appropriate indication for the present technique. The patient is positioned supine with a standard leg holder (Mizuho), allowing full range of motion. To ensure optimal visualization during the procedure, an additional monitor should be placed on the affected side (Fig 2). With the patient under general anesthesia, the surgery begins with a standard knee arthroscopy using anterolateral and anteromedial portals with a 30° arthroscope (Fig 3A). Usually, a tourniquet is not necessary, although it can be used based on visualization. With the knee flexed at 90° on the operating table, the arthroscope is inserted into the posteromedial compartment, passing between the anterior cruciate ligament and the medial femoral condyle, positioned beneath the posterior cruciate ligament. To establish the posteromedial portal, a 70° arthroscope and a 21-gauge needle are used to confirm the precise location, which is the soft spot near the posteromedial margin of the medial femoral condyle (Fig 3B).

**Table 1:** Tips and Tricks for the Procedure Based on Our Experience

Indications
Localized pigmented villonodular synovitis of the posteromedial knee compartment
Surgical steps
Preparation
To ensure optimal visualization, an additional monitor should be placed on the affected side.
Perform standard knee arthroscopy with anterolateral and anteromedial portals.
Insert the scope into the posteromedial compartment between the anterior cruciate ligament and the medial femoral condyle beneath the posterior cruciate ligament.
Confirm the location of the posteromedial portal using a 70° scope and a 21-gauge needle.
The location is the soft spot near the posteromedial margin of the medial femoral condyle.
Create the posteromedial portal.
Enlarge the posteromedial portal to allow the space available for a surgeon’s index finger.
Curettage
Use the punch and scope simultaneously through a single posteromedial portal without irrigation saline.
Use a 3.5-mm shaver and scope simultaneously through the same portal with irrigation.
Use a radiofrequency device and scope simultaneously through the same portal with irrigation.
Confirmation
Complete arthroscopic local clearance through posteromedial and anterolateral portals with or without irrigation.

**Fig 4:**
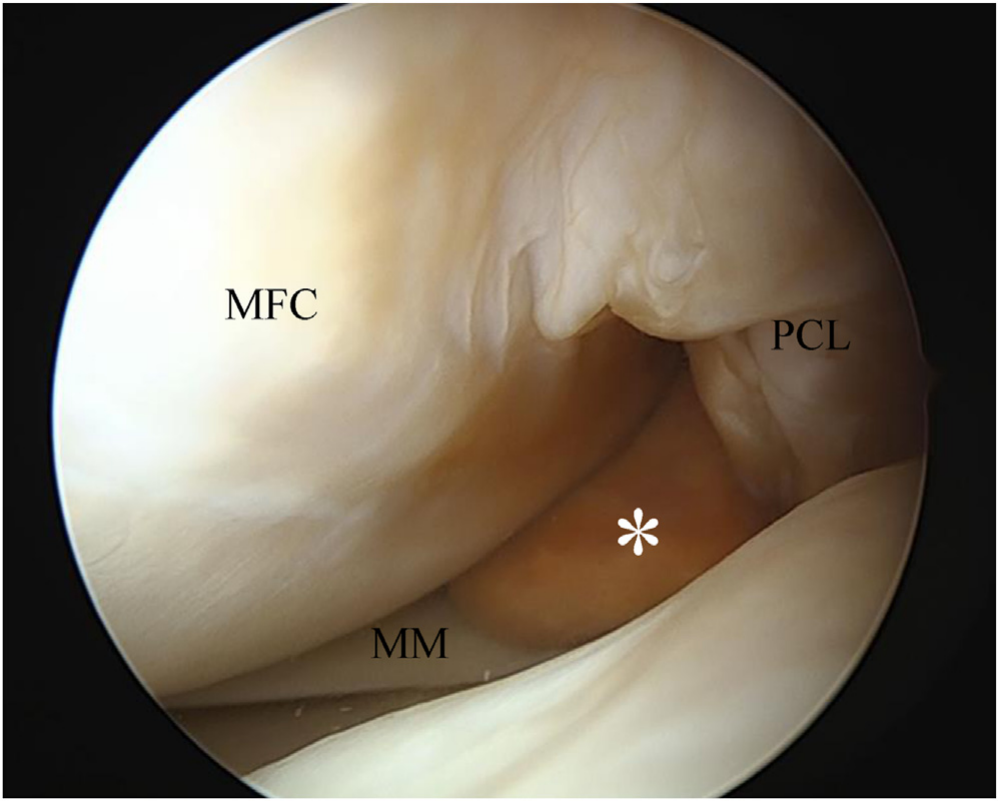
Visualization of localized pigmented villonodular synovitis (white asterisk) in the posteromedial compartment through the standard anterolateral (viewing) portal. (MFC, medial femoral condyle; MM, medial meniscus; PCL, posterior cruciate ligament.)

**Fig 5:**
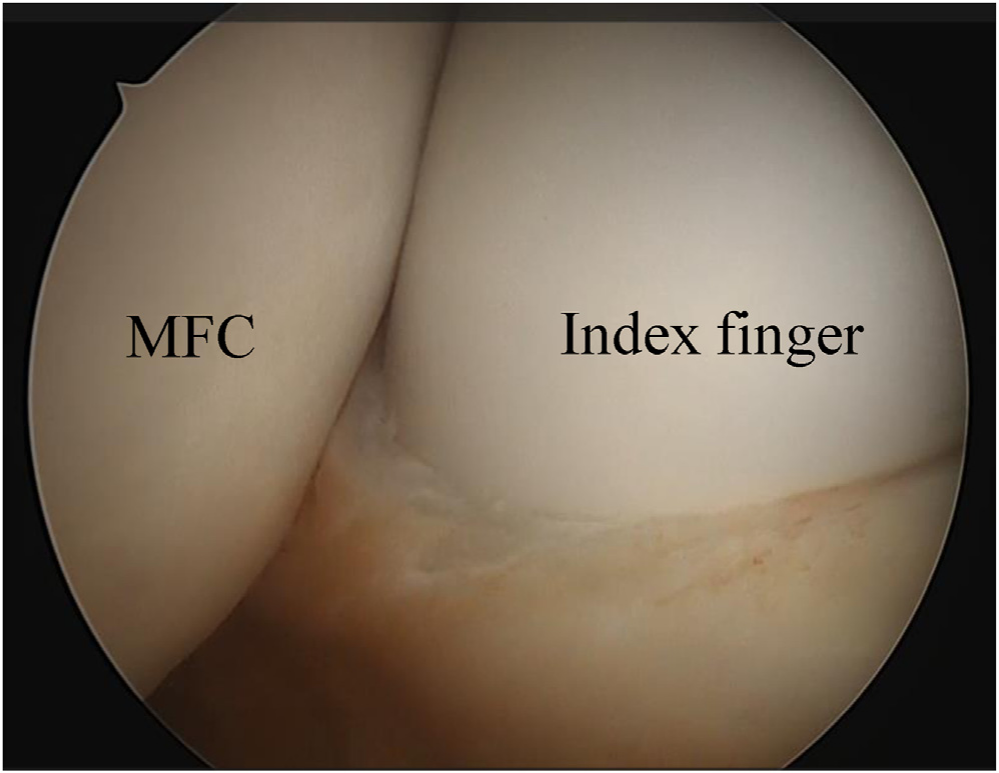
Posteromedial portal is created and then enlarged sufficiently to accommodate the surgeon’s index finger, ensuring adequate space for maneuvering surgical instruments based on the viewing from anterolataral portal after the arthroscope is inserted into the posteromedial compartment, passing between the anterior cruciate ligament and the medial femoral condyle, positioned beneath the posterior cruciate ligament.

**Fig 6:**
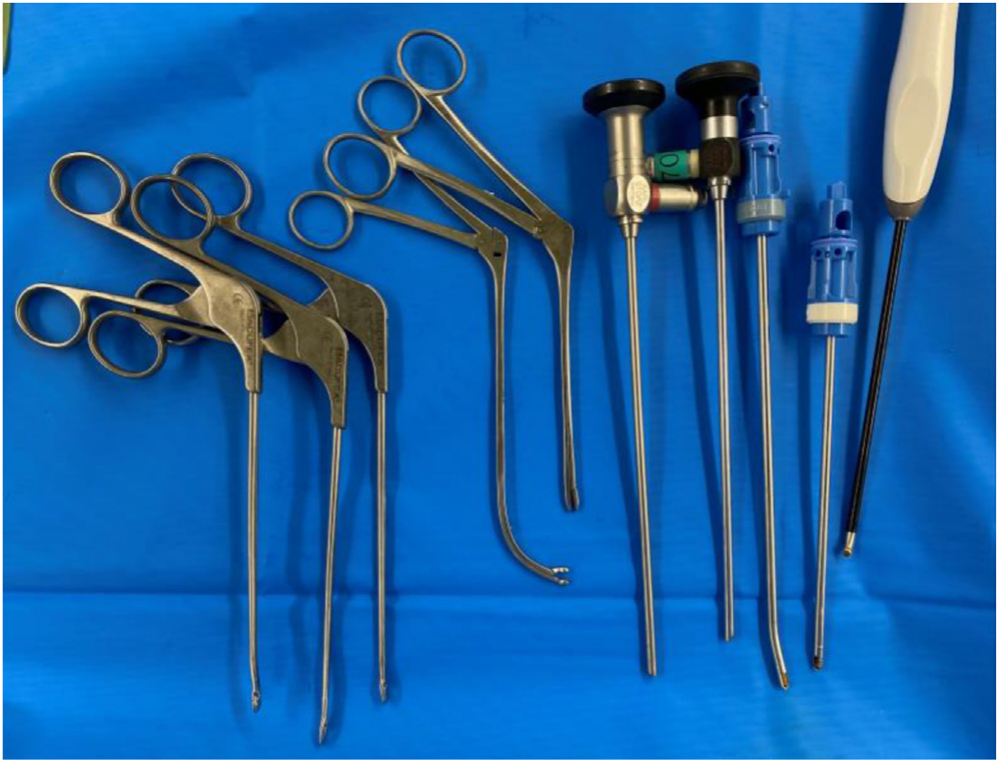
Arthroscopic instruments used for resection, including curved punches and shavers, specifically designed to improve access to posterior synovial lesions.

After assessing the knee joint through the anterolateral portal, PVNS in the posteromedial compartment is detected (Fig 4). Once identified, the posteromedial portal is created and then enlarged sufficiently to accommodate the surgeon’s index finger, ensuring adequate space for maneuvering surgical instruments (Fig 5). To facilitate comprehensive resection, a variety of specialized arthroscopic instruments should be prepared, including curved punches and shavers, which are particularly useful for accessing posterior synovial lesions (Fig 6).

**Fig 7:**
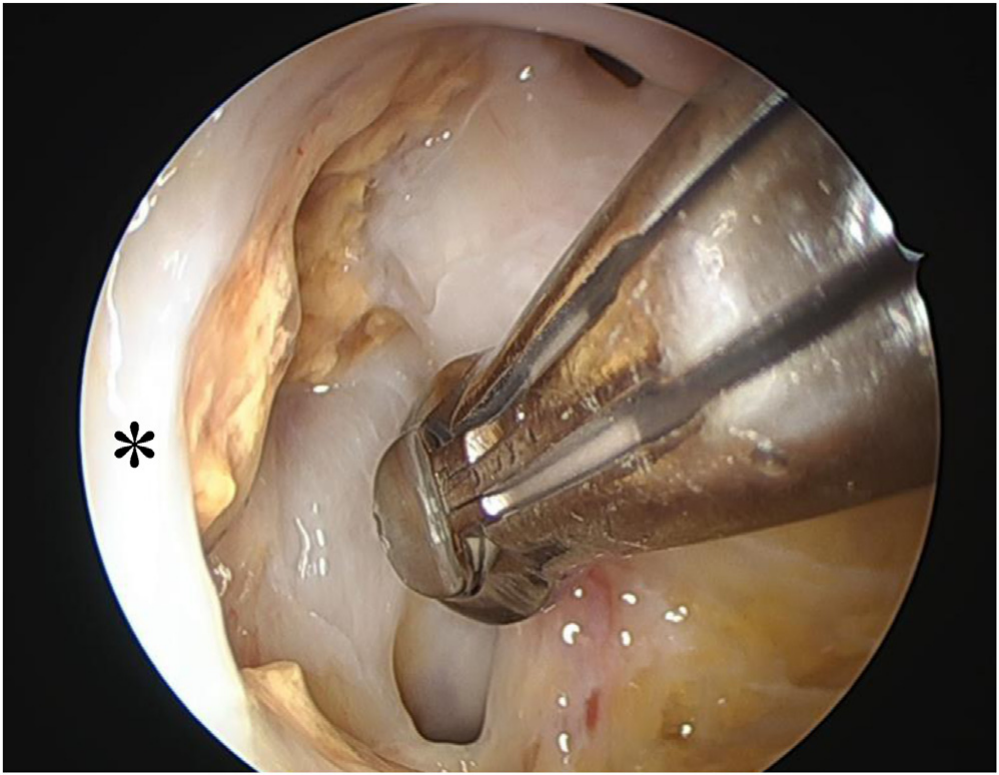
Initial curettage using a punch instrument and simultaneous arthroscopic visualization through the single expanded posteromedial portal, performed without continuous saline irrigation. (Black asterisk: posterior capsule.)

**Fig 8:**
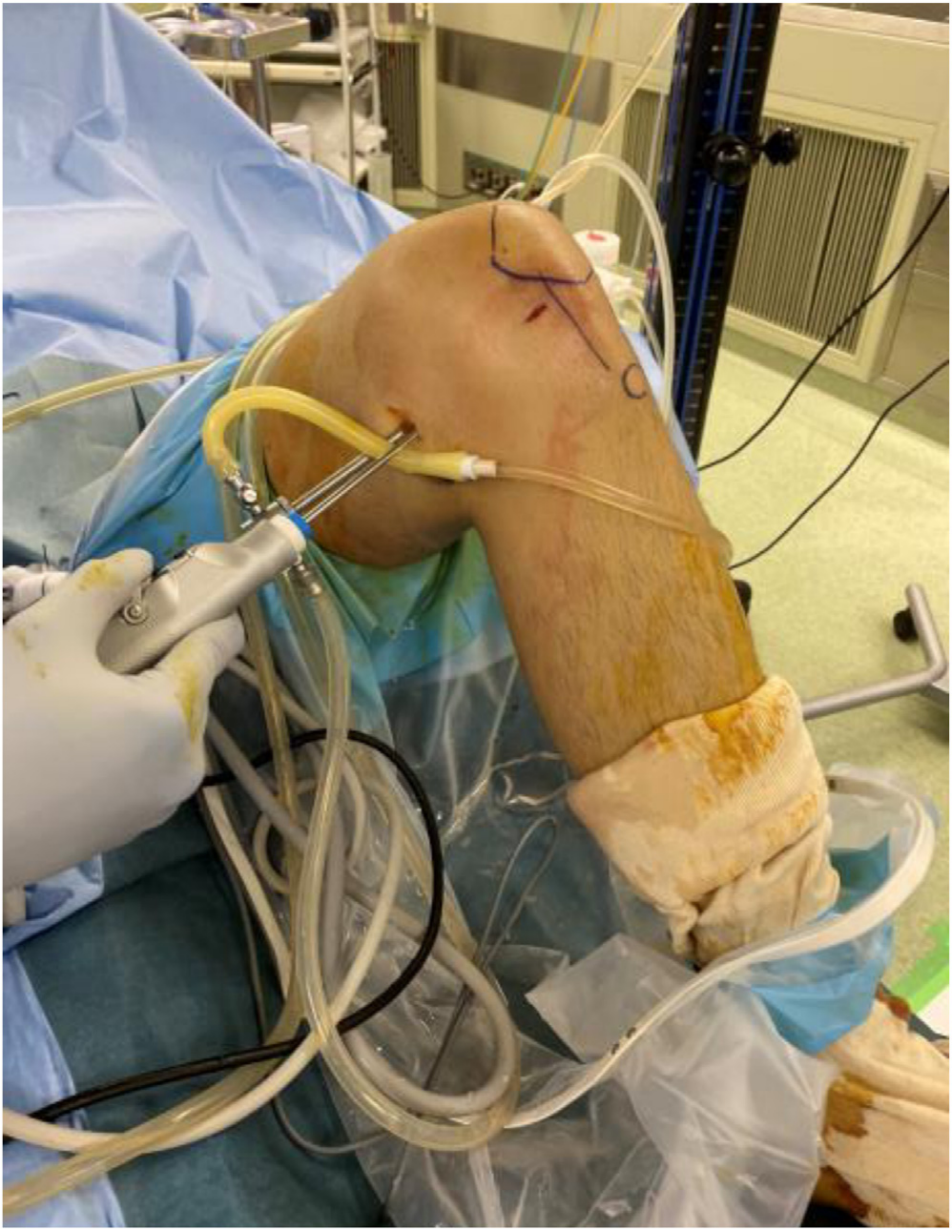
Further debridement using a 3.5-mm shaver (Smith & Nephew Endoscopy) introduced through the posteromedial portal with continuous saline irrigation, ensuring effective removal of residual pigmented villonodular synovitis tissue with the knee flexed at 90° on the operating table. An arthroscope is used simultaneously through the same posteromedial portal.

The resection process starts with initial curettage, where a punch instrument and an arthroscope are used simultaneously through the single posteromedial portal without irrigation (Fig 7). The use of curved punches can be advantageous for excising lesions in areas that are difficult to reach with straight instruments. PVNS tissue can present villous, thread-like structures and globous masses with tan, brown, or red coloration. This is followed by further debridement using a 3.5-mm shaver (Smith & Nephew Endoscopy), introduced through the same portal with continuous saline irrigation, effectively removing residual PVNS tissue (Fig 8). Curved shavers are sometimes helpful in addressing posterior synovial folds and ensuring complete clearance. Finally, a radiofrequency device (Smith & Nephew Endoscopy) is utilized, again through the posteromedial portal with irrigation, to achieve hemostasis and ensure complete resection. To avoid iatrogenic damage, careful curettage is necessary. In particular, indiscriminate shaving of the posterior joint capsule should be avoided.

**Fig 9:**
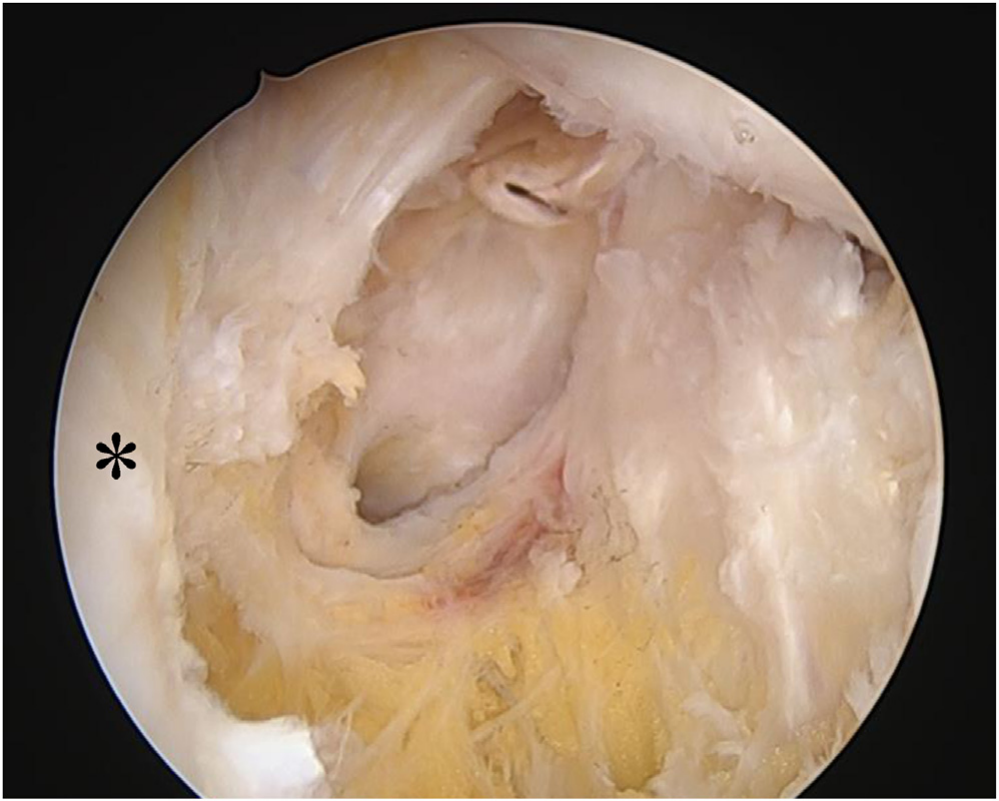
Arthroscopic visualization is performed with or without irrigation through the posteromedial portal, confirming complete clearance of the affected tissue following resection. (Black asterisk: posterior capsule.)

**Fig 10:**
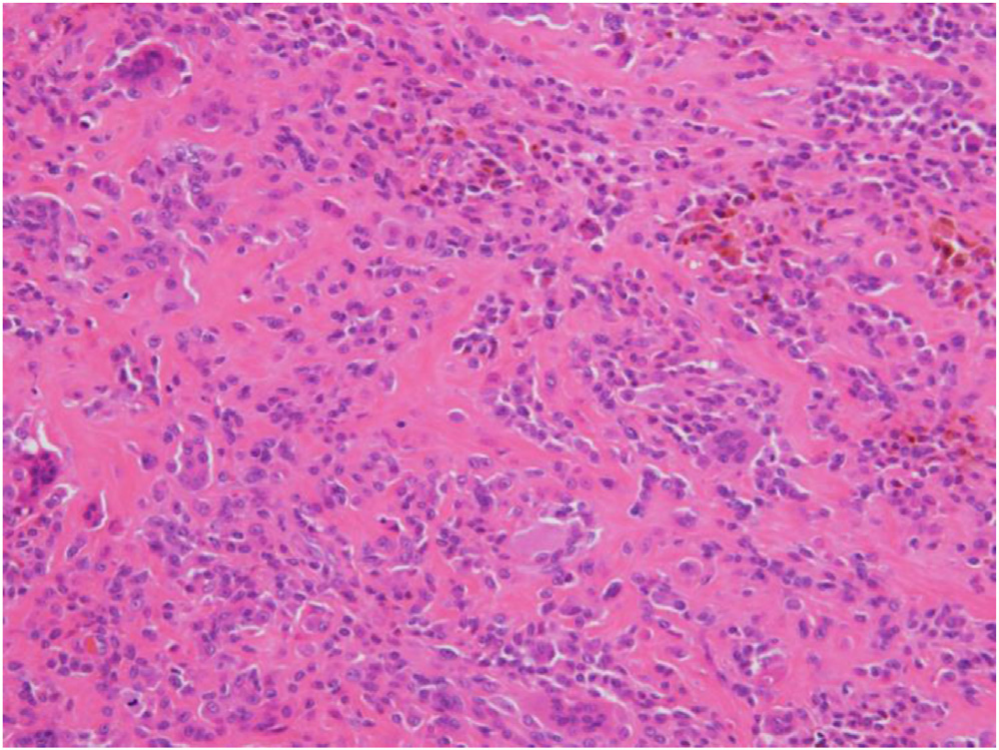
Histopathologic examination confirming the diagnosis of localized pigmented villonodular synovitis, showing characteristic hemosiderin deposition and multinucleated giant cells.

**Fig 11:**
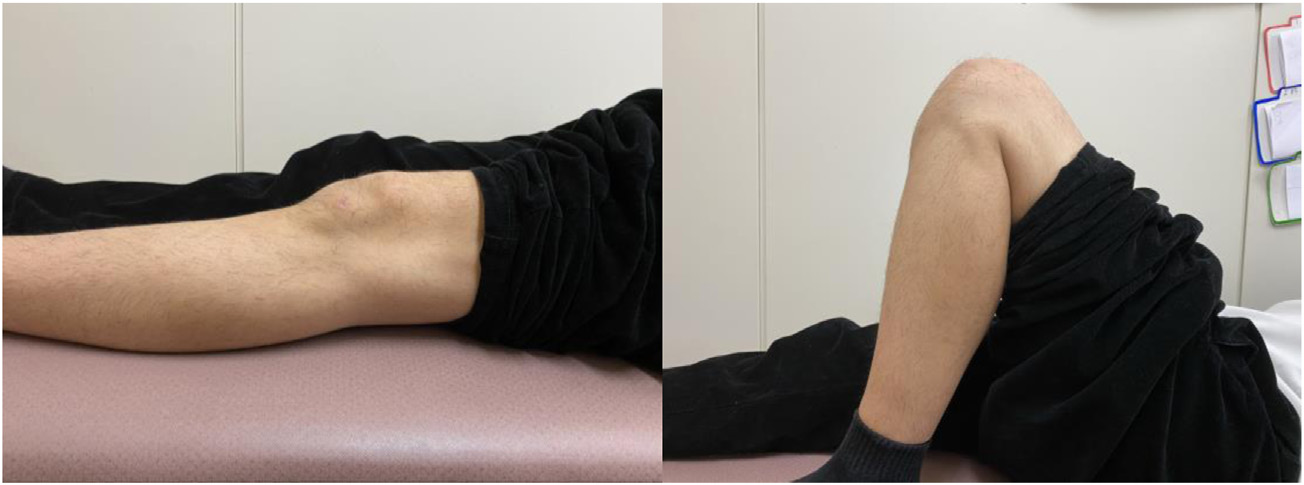
Postoperative clinical image at 3 months showing full functional recovery of the left knee with no range-of-motion limitations or daily activity restrictions.

**Fig 12:**
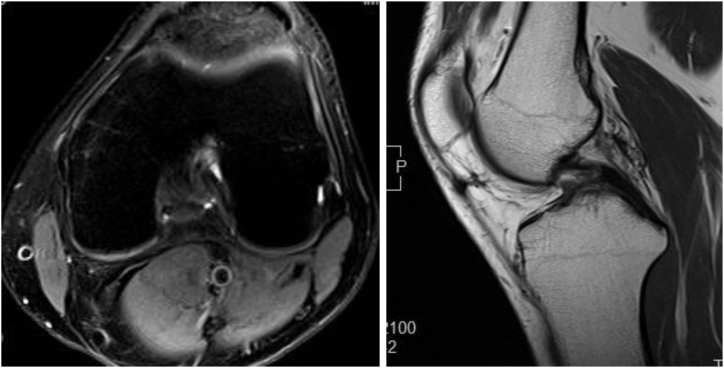
One-year postoperative magnetic resonance imaging of the left knee revealing no evidence of recurrence, indicating the efficacy of the arthroscopic technique in achieving complete resection.

The selection of appropriate instruments is critical for optimizing visualization and ensuring thorough excision of all affected synovium, particularly in anatomically challenging regions of the posterior knee compartment. To confirm the adequacy of the resection, arthroscopic visualization is performed with or without irrigation through both the posteromedial and anterolateral portals, ensuring thorough clearance of the affected tissue (Fig 9). Histopathologic findings are presented in Fig 10. Postoperative knee range of motion and magnetic resonance imaging are shown in Figures 11 and 12, respectively.

## Discussion

Arthroscopic management of localized PVNS in the posterior knee compartment has traditionally been limited by poor visualization and the risk of iatrogenic injury to surrounding neurovascular structures. Our technique aims to overcome these challenges by utilizing an enlarged posteromedial portal, which provides improved access to the lesion while maintaining a minimally invasive approach.

A key advantage of this method is the ability to simultaneously introduce both the arthroscope and surgical instruments through a single portal, allowing for more precise control during curettage (Table 2). This differs from prior approaches requiring both posteromedial and posterolateral portals, which—while providing greater exposure—can increase the complexity of the procedure and the potential for soft tissue trauma.^5^ Additionally, our approach contrasts with methods that attempt lesion excision exclusively from anterior portals, as these have been shown to be associated with a higher risk of incomplete resection due to restricted access to the posterior compartment.^4^ Another important consideration is the recurrence rate of LPVNS. While recurrence rates for diffuse PVNS can be as high as 37.8% for arthroscopic synovectomy,^6^ localized PVNS has been reported to have a much lower recurrence rate (0%-8%) when complete resection is achieved.^7^ Previous studies have shown that incomplete resection is a primary risk factor for recurrence, emphasizing the importance of adequate exposure and careful lesion removal.^6^ Our method enhances visualization and access, reducing the likelihood of leaving residual disease. Despite its advantages, this technique has a limitation. For instance, it is not suitable for cases involving PVNS in the posterolateral compartment, as anatomic constraints make complete resection difficult. Although involvement of the posterior compartment remains relatively uncommon, a posterolateral approach should be considered for PVNS in the posterolateral compartment. Second, careful curettage is necessary to avoid iatrogenic damage, especially for the posterior joint capsule, which contains neurovascular lesions.

**Table 2:** Key Points, Advantages, and Limitations of This Procedure Based on Our Experience

Key points
Enlarge the posteromedial portal
Use the punch and scope simultaneously through the single posteromedial portal without irrigation saline.
Use a 3.5-mm shaver and a radiofrequency device through the same portal with irrigation.
Advantages
Easy confirmation through the same posteromedial portal
Easy procedure for novice surgeons
Safe and easy procedure for the patient
Does not take much time
Limitations
Localized pigmented villonodular synovitis in the posterolateral compartment should not be indicated for this technique.
To avoid iatrogenic damage, careful curettage is necessary. In particular, indiscriminate shaving of the posterior joint capsule should be avoided.

In conclusion, the present modified arthroscopic approach for LPVNS in the posterior knee compartment offers a safe, efficient, and minimally invasive alternative to traditional methods. By enlarging the posteromedial portal, we can achieve improved visualization, better instrument maneuverability, and a lower risk of incomplete excision.

## Declaration of Generative AI and AI-Assisted Technologies in the Writing Process

During the preparation of this work, the authors used the AI-powered text-to-speech software Voice gate for the English narration in the accompanying video due to the authors’ nonnative proficiency in English pronunciation. After using this tool/service, the authors reviewed and edited the content as needed and take full responsibility for the content of the publication.

## Disclosures

All authors (K.H., S.K., T.M., K.K.) declare that they have no known competing financial interests or personal relationships that could have appeared to influence the work reported in this paper.

## Supplementary Data

Video 1 This video shows a modified arthroscopic technique for the resection of localized pigmented villonodular synovitis (LPVNS) in the posterior knee compartment. The patient is positioned supine with a standard leg holder (Mizuho) to allow full knee mobility. An additional monitor is placed on the affected side to optimize visualization. Standard anterolateral and anteromedial portals are established. The posteromedial portal is then created and gradually enlarged to accommodate the surgeon’s index finger, allowing enhanced maneuverability of surgical instruments. Using a curved punch instrument, the initial curettage of the LPVNS lesion is performed under direct arthroscopic visualization. Both the arthroscope and surgical instruments are introduced through the single expanded posteromedial portal, facilitating precise lesion removal. A 3.5-mm shaver (Smith & Nephew Endoscopy) is inserted through the posteromedial portal with continuous saline irrigation to remove residual pigmented villonodular synovitis tissue. A radiofrequency device (Smith & Nephew Endoscopy) is utilized through the posteromedial portal. Final macroscopic visualization is performed through the posteromedial and anterolateral portals to confirm thorough clearance of the affected tissue. One-year follow-up magnetic resonance imaging indicates no evidence of recurrence, showing the efficacy of this modified arthroscopic technique.
